# PLA Melt Stabilization by High-Surface-Area Graphite and Carbon Black

**DOI:** 10.3390/polym10020139

**Published:** 2018-02-01

**Authors:** Luciana D’Urso, Maria Rosaria Acocella, Gaetano Guerra, Valentina Iozzino, Felice De Santis, Roberto Pantani

**Affiliations:** 1 Department of Chemistry and Biology, University of Salerno, Via Giovanni Paolo II 132, 84084 Fisciano (SA), Italy; ldurso@unisa.it (L.D.); macocella@unisa.it (M.R.A.); gguerra@unisa.it (G.G.); 2 Department of Industrial Engineering, University of Salerno, Via Giovanni Paolo II 132, 84084 Fisciano (SA), Italy; viozzino@unisa.it (V.I.); fedesant@unisa.it (F.D.S.)

**Keywords:** poly(lactic acid), carbon black, graphite

## Abstract

Small amounts of carbon nanofillers, specifically high-surface-area graphite (HSAG) and more effectively carbon black (CB), are able to solve the well-known problem of degradation (molecular weight reduction) during melt processing, for the most relevant biodegradable polymer, namely poly(lactic acid), PLA. This behavior is shown by rheological measurements (melt viscosity during extrusion experiments and time sweep-complex viscosity) combined with gel permeation chromatography (GPC) experiments. PLA’s molecular weight, which is heavily reduced during melt extrusion of the neat polymer, can remain essentially unaltered by simple compounding with only 0.1 wt % of CB. At temperatures close to polymer melting by compounding with graphitic fillers, the observed stabilization of PLA melt could be rationalized by scavenging traces of water, which reduces hydrolysis of polyester bonds. Thermogravimetric analyses (TGA) indicate that the same carbon fillers, on the contrary, slightly destabilize PLA toward decomposition reactions, leading to the loss of volatile byproducts, which occur at temperatures higher than 300 °C, i.e., far from melt processing conditions.

## 1. Introduction

It is well known that melt processing of poly(lactic acid) (PLA) (typically conducted at temperatures close to 200 °C) generally leads to degradation, i.e., high molecular weight reduction [1,2,3,4], even in nitrogen atmospheres [2,3,4,5].

Many different approaches have been proposed to achieve PLA melt stabilization [6,7,8,9,10,11,12,13,14]. In particular, stabilization by compounding with commercial antioxidants and water scavengers [6,7], or with chain extenders (i.e., molecules that reconnect polymer chains broken due to moisture at elevated temperatures), such as organic phosphites [8,9,10] or functional polysilsesquioxane microspheres [11], has been described. Additional thermal stabilization approaches involve polymer crosslinking by suitable agents [12,13,14] and polymer-end protection by acetyl groups [15].

Many reports show, on the basis of thermogravimetric analyses (TGA), that several nanofillers, such as clays and organoclays [16,17,18,19,20,21], silica [22,23], lignin [24], and silk [25] and cellulose [26] nanocrystals, stabilize PLA with respect to decomposition reactions, leading to loss of low-molecular-weight byproducts, which occur at temperatures higher than 300 °C (i.e., very far from melt processing conditions). In recent years, many papers have been published on PLA composites with graphite-based nanofillers [27,28,29,30,31,32,33,34,35,36,37,38,39,40,41,42,43,44,45,46] and carbon black [47,48,49,50]. These papers report significant improvements in PLA’s physical (mainly electrical) properties with only a small amount of filler. Some of these papers report that some carbon fillers, like other nanofillers [16,17,18,19,20,21,22,23,24,25,26], can stabilize PLA with respect to decomposition reactions, leading to volatile byproducts at a temperature higher than 300 °C [27,29,32,34,43,44,45,46,48,49]. For instance, the addition of exfoliated graphite increases of 5–15 °C decomposition temperatures corresponding to a 5% weight loss (*T*_d,5%_) [27,29,32].

Stabilization of the polymer toward decomposition reactions leading to volatile byproducts at high temperatures (for PLA for T > 300 °C) does not assure maintenance of polymer molecular weight during melt processing (for PLA at T ≈ 200 °C). In fact, some fillers (e.g., clays) that stabilize PLA toward high-temperature decomposition reactions destabilize PLA toward degradation reactions, leading to more pronounced polymer molecular weight reductions, in melt processing conditions [51,52,53,54,55,56]. In this respect, it is also worth adding that the temperatures corresponding to a 10% weight loss (*T*_d,10%_) for PLA and isotactic polypropylene (PP) were evaluated as 320 °C and 270 °C, while PP was incomparably more stable than PLA during melt processing [51].

In this paper, the influence of different graphite-based fillers—A low-surface-area graphite (LSAG), a high-surface-area graphite (HSAG), and a carbon black (CB)—On the stability of PLA melt at different temperatures is reported. In particular, PLA stability during melt processing (at 200 °C) was studied via melt viscosity measurements during extrusion, via evaluations of molecular weight distributions of the extruded unfilled and filled samples with gel permeation chromatography (GPC), and via rheological measurements (time sweep-complex viscosity). PLA stability at higher temperatures (above 300 °C), where decomposition reactions lead to loss of volatile byproducts, was studied via TGA.

It was found that the considered graphite-based fillers do not improve (or even slightly reduce) PLA thermal stability above 300 °C, i.e., they marginally affect high temperature decomposition reactions, leading to low-molecular-mass byproducts. However, very small amounts (as low as 0.1 wt %) of high-surface-area graphite and of carbon black, are sufficient to inhibit degradation reactions, leading to molecular weight reductions without a loss of volatile byproducts, which occur during processing at 200 °C.

## 2. Experimental

In this work, a commercial grade of PLA produced by NatureWorks (Minnetonka, MN, USA) with the trade name of 4032D was adopted. This PLA grade has a d-enantiomer content of approximately 2% and a maximum degree of crystallinity of about 45%. A thermal and rheological characterization of the material can be found in the literature [57,58,59,60].

The material was dried at 60 °C under vacuum overnight before any processing and testing operation.

Primary Synthetic Graphite TIMREX^®^ SFG6 with a low surface area (LSAG, of about 17 m^2^/g), an average particle size of 6 μm, and a carbon amount of 99.6% was provided by Timcal Graphite & Carbon (Bodio, Switzerland). Synthetic Graphite TC 307 with a high surface area (HSAG, of about 352 m^2^/g), a primary particle size less than 1 μm, a carbon amount of 99.92%, and a high shape anisotropy of the crystallites [61] was purchased from Asbury Graphite Mills, Inc (Asbury, NJ, USA). The used carbon black sample (CB) of grade N660, with a surface area of 33 m^2^/g and a particle size around 49–60 nm, was purchased from Cabot Company (Boston, MA, USA).

Wide-angle X-ray diffraction (WAXD) patterns were obtained by an automatic Bruker D8 Advance diffractometer (Bruker Corp, Billerica, MA, USA), in reflection, at 35 kV and 40 mA, using nickel-filtered Cu Kα radiation (0.15418 nm). The *d* spacings were calculated using Bragg’s law, and the observed integral breadths (β_obs_) were determined by a fit with a Lorentzian function of the diffraction patterns. The instrumental broadening (β_inst_) was also determined by fitting of a Lorentzian function to line profiles of a standard silicon powder 325 mesh (99%). The corrected integral breadths of the 002 peak were determined by subtracting the instrumental broadening of the closest silicon reflection from the observed integral breadths, β = β_obs_ − β_inst_. The correlation lengths (*D*) were determined using Scherrer’s equation:

(1) $$D=\frac{K\lambda }{\beta \;\cos\theta }$$

where *λ* is the wavelength of the incident X-rays and θ the diffraction angle, assuming the Scherrer constant *K* = 1.

Melt compounding was carried out by a microcompounder (twin screws, counter-rotating, Haake Mini-lab II, Thermo Fisher Scientific, Schwerte, Germany). Thanks to a backflow channel and a bypass valve, it is possible to define the residence time in the microcompounder. Furthermore, this device allows one to estimate the viscosity as the material is compounded in the back flow channel that is a rectangular slit with two pressure transducers [62,63]. The tests were conducted at a temperature of 200 °C and a screw rotation speed of 100 rpm. Under these conditions, the estimated shear rate at which the viscosity was calculated is about 350 s^−1^. After 15 min, the bypass valve is opened and the compound is extruded.

The zero-shear rate viscosity of a polymer can be related to the molecular weight of the polymer by the following equation:

(2) $$\eta =c\;Mw^{a}$$

where *a* is an exponent whose value is generally accepted to be 3.4 [64], and *c* is a parameter that depends on temperature. According to Equation (2), due to the exponent *a*, the viscosity is extremely sensitive to changes in molecular weight, so rheological measurements are an extremely powerful means of assessing the degradation in the molten state.

The melt compounding was carried out for 15 min (900 s) at 200 °C. The materials were then taken from the microcompounder and used for the subsequent analysis: GPC and rheology.

GPC measurements were conducted by a Waters Breeze GPC system (Waters, Milford, MA, USA), equipped with a refractive index (RI) detector, by using a set consisting of four Styragel HT columns with (102, 103, 104, and 105 Å pore size) and 10 µm (particle size). Tetrahydrofuran, THF, was used as eluent at 35 °C at a flow rate of 1.0 mL·min^−1^. The calibration curve was established with polystyrene standards.

Time sweep experiments were performed by means of a Haake Mars II (Thermo Scientific) rotational rheometer in a plate–plate configuration (*D* = 20 mm) under a dry nitrogen atmosphere. A constant stress of 100 Pa and a frequency of 1 rad/s were applied during the tests. In this condition, all measurements were carried out within the linear response domain and within the Newtonian plateau for all materials. The tests were carried out at a temperature of 200 °C for about 3 h.

TGA analyses were conducted with a TG 209 F1, manufactured by Netzsch Geraetebau (Selb, Germany), with a heating rate of 10 K/min under an N_2_ flow.

DSC scans were conducted at a heating rate of 10 K/min. and the results are reported in the supplementary material (Figure S1).

## 3. Results and Discussion

### 3.1. WAXD Characterization of Carbon Fillers

**Figure 1:**
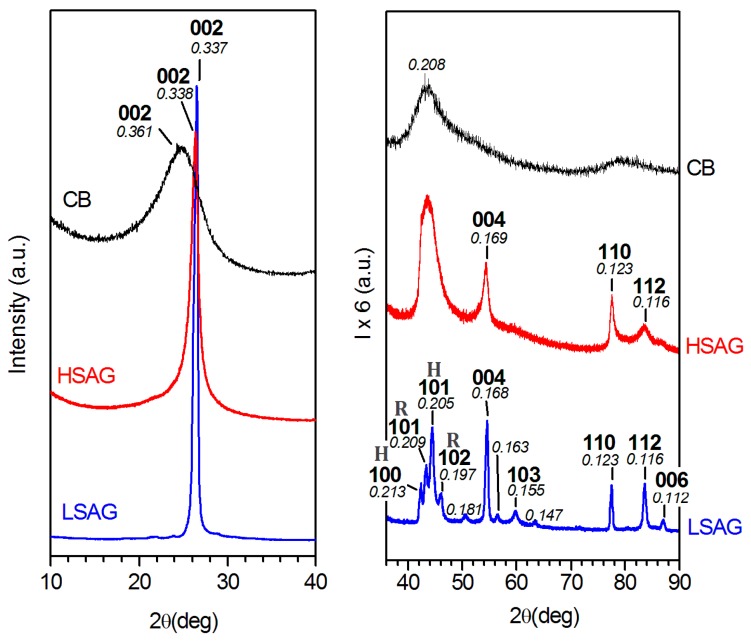
WAXD patterns (Cu Kα), as collected by an automatic powder diffractometer, of used carbon fillers: LSAG (lower blue curve); HSAG (intermediate red curve); CB (upper black curve). H and R labels refer to reflections being specific of hexagonal and rhombohedral phases, respectively.

WAXD patterns, as collected by an automatic powder diffractometer, of the used low-surface-area (blue curve) and high-surface-area (red curve) graphites are compared in Figure 1. It is immediately apparent that their crystalline structures are largely different. In particular, LSAG exhibits a much more ordered structure, with a large number of narrow diffraction peaks. These peaks can be easily indexed by assuming the presence of both hexagonal or rhombohedral phases [65,66]. Particularly informative is the pattern region with 42° < 2θ_Cu Kα_ < 46°, where four well defined peaks are present, with two peaks at *d* = 0.213 nm and *d* = 0.205 nm indexed as (100) and (101) reflections of the hexagonal phase and two peaks at *d* = 0.209 nm and *d* = 0.197 nm indexed as (101) and (102) reflections of the rhombohedral phase. The fraction of rhombohedral modification is approximately 30%, as derived by comparing the integrated intensities for the above cited hexagonal and rhombohedral peaks.

HSAG exhibits a much more disordered structure, with a strongly reduced number of diffraction peaks. In particular, besides (00l) reflections and the in-plane 110 reflection at *d* = 0.123 nm, only a broad diffraction halo is present that is roughly centered at *d* = 0.208 nm. This clearly indicates the occurrence of a turbostratic graphite with a nearly complete disorder in the relative position of parallel graphitic layers [61,65,67].

For both LSAG and HSAG, the distance between parallel graphitic layers is equal to *d* = 0.337 nm, while the corresponding correlation length perpendicular to the graphitic planes (as evaluated by breadths of the 002 peak) is much lower for HSAG, with *D*_⊥__,HSAG_ = 12 nm and *D*_⊥__,LSAG_ = 26 nm.

For the sake of comparison, the WAXD pattern of the used carbon black is also shown in Figure 1. As discussed in detail in a recent paper [68], WAXD patterns of CB (as well as of oxidized CB, oCB) samples suggest that they are prevailingly constituted by a disordered spatial arrangement of highly defective structural layers with short in-plane correlation lengths (2–3 nm). This was confirmed by the ability of oCB to form ordered intercalation compounds [68].

### 3.2. Melt Compounding of PLA in the Presence of Different Kinds and Amounts of Carbon Fillers

**Figure 2:**
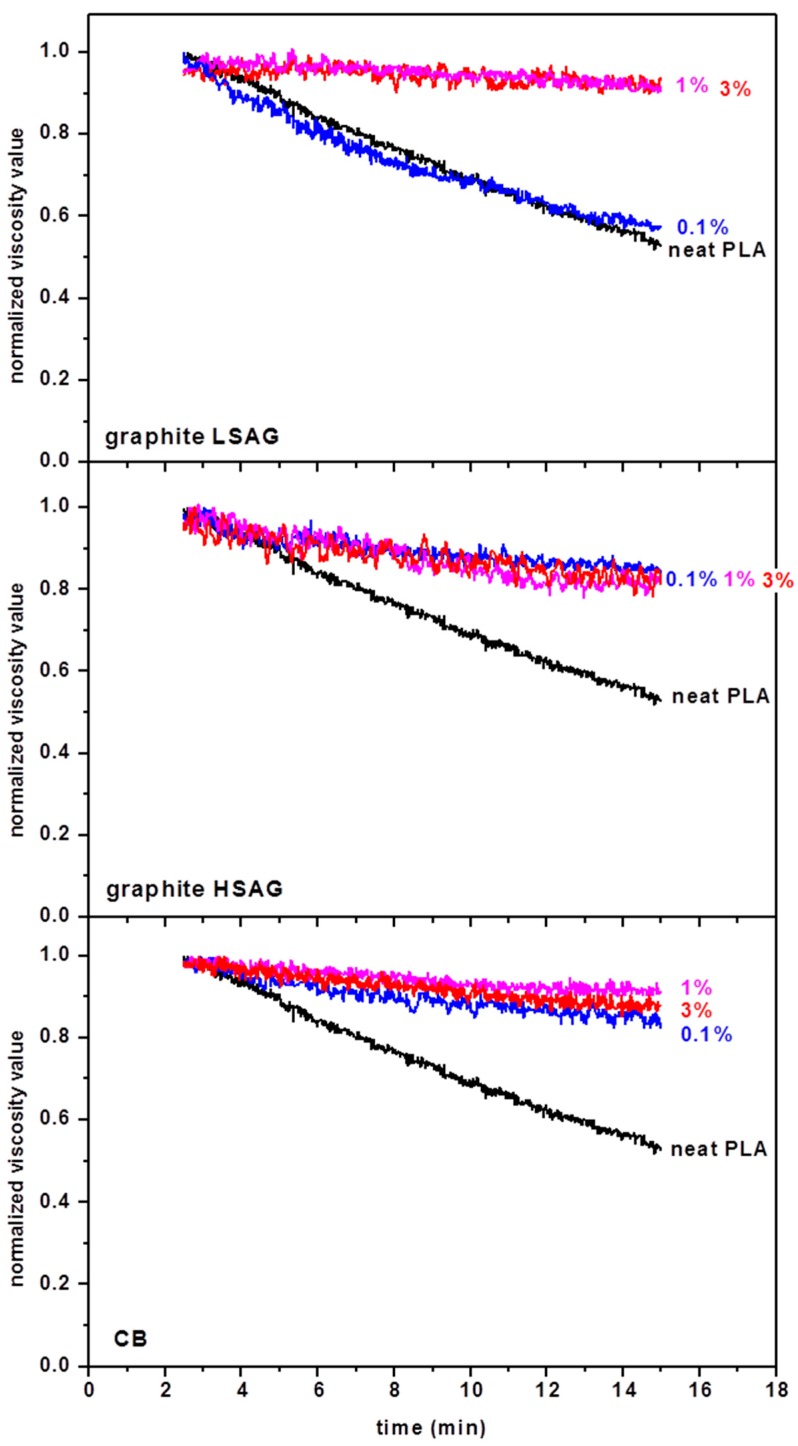
Time evolution of normalized viscosity (with respect to the initial value) in the microcompounder at *T* = 200 °C and 100 rpm. The estimated value of shear rate is about 350 s^−1^.

Viscosity values measured during compounding are reported in Figure 2. The data are normalized with respect to the initial values measured for each material, so that all values start from 1. The curve which refers to the neat PLA is reported for comparison in all the plots of Figure 2. It can be seen that, in agreement with that reported in the literature [5,69], the viscosity of pure PLA during compounding immediately starts to reduce significantly, such that viscosity becomes about one half of the starting value after about 15 min. According to Equation (2), this reduction suggests a reduction of about 20% in the molecular weight of the material.

All the fillers adopted, with the exception of graphite LSAG at the lowest used percentage of 0.1%, introduce a significant stabilizing effect, such that the reduction of viscosity with time is limited to about 10%. For graphite HSAG and CB, this effect is reached already for filler contents of 0.1%, which is an extremely significant result for PLA processing.

**Figure 3:**
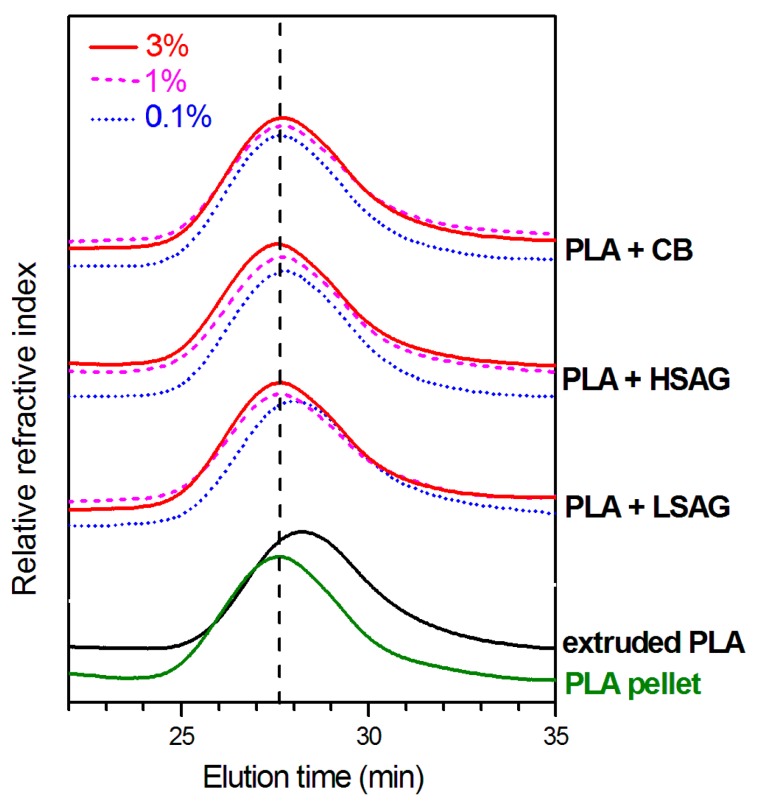
GPC curves in THF at 35 °C of the PLA pellet (green) of extruded PLA (black) as well as of extruded PLA compounds, as obtained by processes whose viscosity reduction is shown in Figure 2. Compounds contain 0.1 wt % (dotted lines), 1 wt % (dashed lines), and 3 wt % (continuous lines) of different graphitic fillers: LSAG, HSAG, and CB.

GPC curves of PLA pellet and of extruded PLA compounds, with different kinds and amounts of carbon fillers, are shown in Figure 3.

Elution times of GPC curves of PLA are definitely lower before extrusion (green lowest curve in Figure 3) than after extrusion (black curve in Figure 3). This confirms that, as generally observed for PLA, extrusion processes lead to a substantial polymer degradation. As shown in Table 1, GPC curves indicate a reduction of about 25% of the initial number-average molecular weights (*M*n and *M*w). This is consistent with a reduction in viscosity of about 40%, in agreement with the results shown in Figure 2.

GPC curves of the extruded PLA compounds indicate that, for all the considered carbon fillers, a concentration of 3 wt % (continuous lines in Figure 3) is able to eliminate the adverse effect of the considered PLA processing on molecular mass. Moreover, for HSAG and CB, a concentration as low as 0.1 wt % is sufficient to stabilize PLA to the molecular mass of the virgin pellet (dotted curves in Figure 3, 7th and 10th columns in Table 1). Again, these results are consistent with rheological data reported in Figure 2.

**Figure 4:**
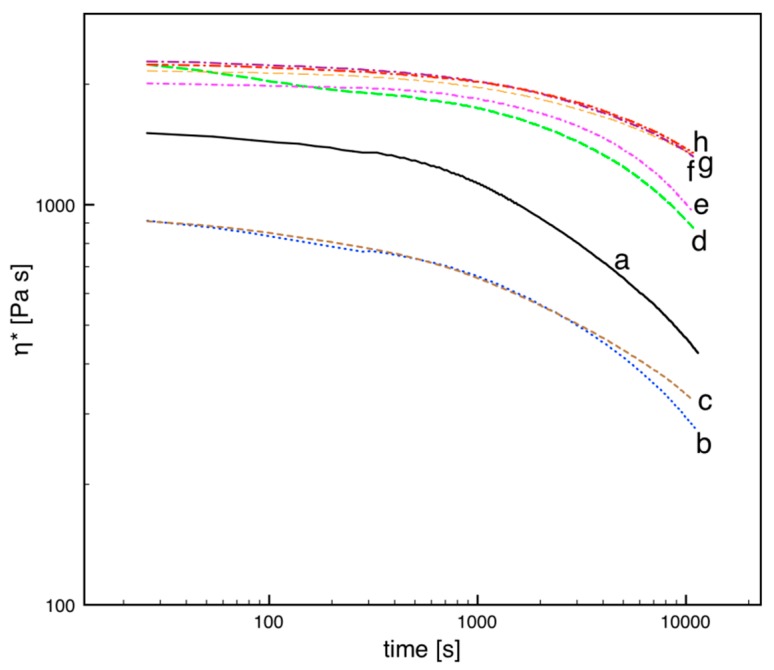
Time sweep-complex viscosity for the virgin pellet (**a**) and for extruded PLA samples after microcompounding: (**b**) neat and with (**c**) 0.1 wt % of LSAG; (**d**) 1 wt % of LSAG; (**e**) 0.1 wt % of HSAG; (**f**) 1 wt % of HSAG; (**g**) 0.1 wt % of CB; (**h**) 1 wt % of CB. Experimental conditions: *T* = 200 °C, *f* = 1 rad/s, plate–plate, gap = 200 µm.

The PLA pellet and the extruded PLA compounds were analyzed by time sweep rheological tests. The results are reported in Figure 4 and show even more clearly the thermal stabilization effect of carbon fillers. These measurements, which were carried out for a very long time (about 3 h) indicate a degradation at high temperature after the microcompounding step. Under these conditions, it is possible to discriminate between stabilization effects of HSAG and CB, which would appear to be very similar on the basis of the viscosity measurements of Figure 2 as well as on the basis of the GPC data of Figure 3 and Table 1, which indicate the effect of the microcompounding step.

**Figure 5:**
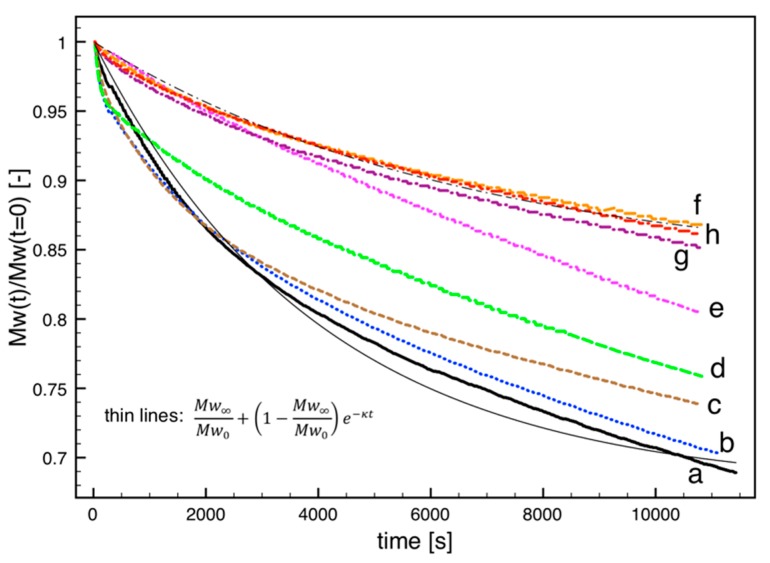
Time sweep-complex viscosity for the virgin PLA pellet (**a**) and for extruded samples after microcompounding: (**b**) neat and with (**c**) 0.1 wt % of LSAG; (**d**) 1 wt % of LSAG; (**e**) 0.1 wt % of HSAG; (**f**) 1 wt % of HSAG; (**g**) 0.1 wt % of CB; (**h**) 1 wt % of CB. Experimental conditions: *T* = 200 °C, *f* = 1 rad/s, plate–plate, gap = 200 µm.

The viscosity evolution reported in Figure 4 can be interpreted in terms of molecular weight according to Equation (2). The results are reported in Figure 5.

The initial molecular weight, *M*w(*t* = 0), indicates the value after the microcompounding step for all the samples except the PLA in pellet. The stabilizing effect of the fillers is clearly evidenced: the virgin polymer (both neat and extruded) presents a reduction of about 30% in molecular weight during the 3 h of the test at 200 °C. All considered carbon fillers indicate a slower decrease in *M*w, with an effect that generally depends on concentration: an increase from 0.1% to 1% induces a slower degradation. For CB, 0.1% and 1% induce the same effect and allow for a reduction of just 10% of *M*w during the test.

The obtained results appear to be nearly independent of the surface area of carbon black. In fact, similar PLA melt stabilization was obtained using a different carbon black sample, exhibiting a definitely higher surface area (N110 with surface area of 150 m^2^/g).

The observed polyester melt stabilization by HSAG and mainly by carbon black could be explained by the removal of traces of water from the melt, thus strongly reducing the hydrolysis of polyester bonds.

Indeed, on assuming that water is the only reason for molecular chain scission, namely assuming that hydrolysis is the only degradation mechanism (which is surely a simplification of more complex mechanisms taking place at high temperatures), one can relate the amount of water to the amount of carboxylic end groups according to the following equations:

(3) $$\frac{dCa}{dt}=-\frac{dCc}{dt}\Rightarrow Ca=\left(Cc_{0}+Ca_{0}\right)-Cc=Cc_{\infty }-Cc$$

in which *Ca* is the concentration of water inside the samples and *Cc* is the concentration of carboxylic end groups. The subscripts 0 and *∞* indicate the initial concentrations (at *t* = 0) and the final situation in which water molecules completely disappeared, respectively.

Considering that the concentration of carboxylic end groups is related to the concentration of polymeric chains

(4) $$Cc=\frac{\rho }{Mn}$$

one simply obtains

(5) $$Ca=\frac{\rho }{Mn_{\infty }}\left(1-\frac{Mn_{\infty }}{Mn}\right)$$

and finally

(6) $$Ca_{0}=\frac{\rho }{Mn_{\infty }}\left(1-\frac{Mn_{\infty }}{Mn_{0}}\right).$$

Assuming that, at least during early stages of degradation, the polydispersity index (*M*w*/M*n) can be considered to be constant, as also confirmed by the GPC data reported in Table 1, the data reported in Figure 5 can be considered to refer to *M*n*/M*n_0_ during melt degradation. Those time evolutions can be fitted by a simple exponential curve, which can provide an estimate of the molecular weight at long times. For the samples a and h in Figure 5, the exponential fitting is reported. On knowing the density (1.2 g/cm^3^) and the initial molecular weight from GPC data reported in Table 1, the initial water concentration is easily calculated. For the sample a (neat, virgin material), *Ca*_0_ is 4.7 mol/m^3^, corresponding to about 70 ppm. For the sample h (1 wt % of CB), *Ca*_0_ is 2 mol/m^3^, corresponding to about 30 ppm. The indication provided by the simplified model described above is that the amount of water taking part in the hydrolysis can be substantially reduced (of a factor 2 or more) by adding the fillers analyzed in this work.

This water absorption possibly occurs by specific interactions between water and oxidized groups on carbon surfaces [70].

### 3.3. Weight Loss in TGA Experiments of PLA in the Presence of Carbon Fillers

**Figure 6:**
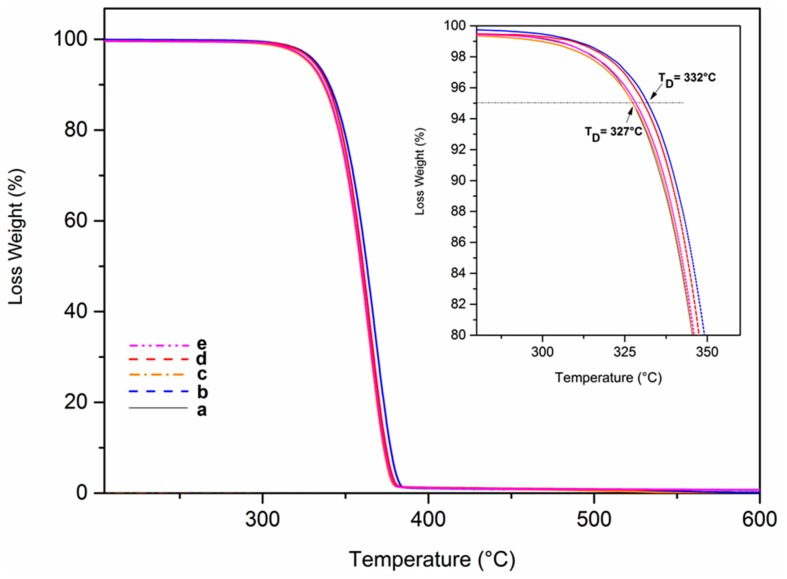
TGA scans for the virgin PLA pellet (**a**) and for extruded samples after microcompounding: (**b**) neat and with (**c**) 0.1 wt % of LSAG; (**d**) 0.1 wt % of HSAG; (**e**) 0.1 wt % of CB. Experimental conditions: a heating rate of 10 K/min under an N_2_ atmosphere.

TGA scans of the extruded compounds indicate that PLA thermal stability, expressed in terms of weight loss, is slightly reduced by compounding with all the considered carbon fillers. Just as an example, TGA scans with a heating rate of 10 K/min for the PLA pellet, for the neat PLA-extruded sample, and for the extruded nanocomposites with 0.1 wt % of filler concentration are compared in Figure 6.

An increase in the decomposition temperature, e.g., corresponding to a 5% of weight loss (*T*_D,5%_), is observed going from the virgin (*T*_D,5%_ = 327 **°**C) to the extruded neat (*T*_D,5%_ = 332 °C) sample, although the latter has undergone a remarkable molecular weight reduction (GPC data of Table 1). Moreover, compounding with all considered carbon fillers leads to small decreases of *T*_D,5%_, mainly for CB (*T*_D,5%_ = 328 °C).

The results of Section 3.2 clearly indicate that carbon fillers stabilize PLA toward degradation reactions occurring at temperatures close to 200 °C, not far from PLA’s melting temperature, which leads to a reduction in the molecular masses, without any loss of volatile degradation products. The TGA results of this section indicate that the same carbon fillers, on the contrary, slightly destabilize PLA toward decomposition reactions occurring at temperatures higher than 300 °C, which leads to a loss of volatile byproducts.

## 4. Conclusions

Different carbon fillers, specifically low- and high-surface-area graphite as well as carbon black, have been tested as possible stabilizers of PLA melt. 

Melt viscosity measurements during extrusion processes and GPC experiments on the corresponding extruded samples show a remarkable PLA melt stabilization by all the considered nanofillers, at a temperature just above melting. In particular, melt stabilization leads to degradation reactions, leading to molecular weight reduction without any weight loss. For instance, for extrusions conducted at 200 °C, neat PLA exhibits a molecular weight reduction of about 25% while, by compounding with only 0.1 wt % of HSAG or of CB, PLA’s molecular weight remains unaltered.

Time sweep-complex viscosity measurements, as carried out at 200 °C for about 3 h, confirm the ability of carbon nanofillers to stabilize PLA toward degradation reactions. In fact, PLA’s molecular weight was reduced by about 30% in the virgin polymer (both neat and extruded), while it was reduced by amounts in the range 25–10% in the considered carbon composites. These measurements show that CB most effectively slowed down PLA degradation, for which a content of only 0.1 wt % leads to a molecular weight reduction close to 10%, much lower than that of the neat PLA (30%). 

TGA analyses indicate that the considered carbon fillers, on the contrary, slightly destabilize PLA toward decomposition reactions, leading to a loss of volatile byproducts, which occur at temperatures higher than 300 °C, i.e., far from melt processing conditions.

The observed PLA stabilization by carbon fillers, at temperatures suitable for melt processing, can be explained by scavenging traces of water from the melt, which reduces the hydrolysis of polyester bonds.

In summary, PLA compounding with very small amounts (even 0.1 wt %) of HSAG and CB lead to remarkable PLA stabilization toward reactions, leading to molecular weight reduction. This contributes to the solution of the well-known problem of PLA degradation during processing. The same carbon nanofillers can also be effective in reducing degradation during processing for other polyesters that are sensitive to hydrolysis in the melt state.

## Figures and Tables

**Table 1:** Number average molecular weight (*M*n), weight average molecular weight (*M*w), and polydispersity index (PDI) as evaluated by GPC curves, for PLA pellet and extruded compounds with LSAG, HSAG, and CB. The evaluated variance is of ±3 kDa.

		Extruded PLA Samples									
	PLA Pellet	PLA Neat	LSAG			HSAG			CB		
			0.1%	1%	3%	0.1%	1%	3%	0.1%	1%	3%
*M*n [kDa]	120 ± 3	88	95	120	120	120	121	120	121	121	120
*M*w [kDa]	195 ± 3	146	165	195	196	190	194	196	192	193	193

## Supplementary Materials

The following are available online at http://www.mdpi.com/2073-4360/10/2/139/s1, Figure S1: DSC heating scans of the extruded PLA and PLA compounded with 0.1wt % of HSAG, LSAG and CB.
