# Supplementary material for: PLA Melt Stabilization by High-Surface-Area Graphite and Carbon Black
Source: Polymers (Basel). 2018 Feb 1;10(2):139. doi: 10.3390/polym10020139 (PMC6415102; doi:10.3390/polym10020139)
Supplement: Supplementary file 1 [file polymers-10-00139-s001.pdf]

Supporting Information

PLA melt stabilization by high-surface-area graphite and carbon black

Luciana D'Urso [1], Maria Rosaria Acocella [1] , Gaetano Guerra [1] , Valentina Iozzino [2], Felice De Santis [2] and Roberto Pantani [2,*]

[1] Department of Chemistry and Biology, University of Salerno, Via Giovanni Paolo II 132, 84084 Fisciano (SA), Italy
[2] Department of Industrial Engineering, University of Salerno, Via Giovanni Paolo II 132, 84084 Fisciano (SA), Italy
* Correspondence: rpantani@unisa.it; Tel.: +39-089964141

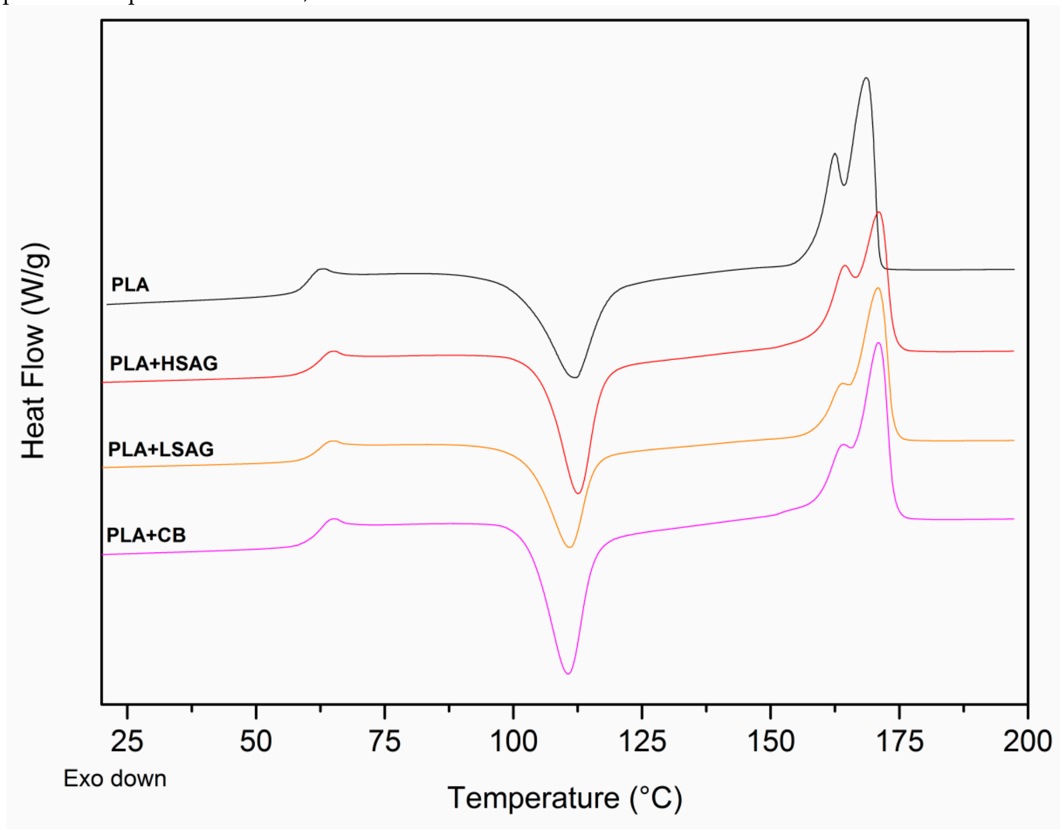

Figure S1. DSC heating scans of the extruded PLA and PLA compounded with 0.1wt % of HSAG, LSAG and CB.

The DSC scans at heating rate of 10 K/min of the extruded samples, neat or compounded with 0.1wt% of the three considered carbon fillers, are shown in Fig.S.1. These scans do not show significant variations in thermal transitions depending on the filler kind. Similar behavior is observed for different content of the same carbon fillers (at least up to 3wt%). In particular, cold crystallization and melting peak temperatures for neat PLA are about 112°C and 169°C, respectively.
